# Characterization and preventability of adverse drug events as cause of emergency department visits: a prospective 1-year observational study

**DOI:** 10.1186/s40360-019-0297-7

**Published:** 2019-04-27

**Authors:** Ivan Lo Giudice, Eleonora Mocciaro, Claudia Giardina, Maria Antonietta Barbieri, Giuseppe Cicala, Maria Gioffrè-Florio, Giuseppe Carpinteri, Aulo Di Grande, Edoardo Spina, Vincenzo Arcoraci, Paola Maria Cutroneo

**Affiliations:** 1 Department of Clinical and Experimental Medicine, University of Messina, Via Consolare Valeria, 98125 Messina, Italy; 2 Department of Emergency Medicine, University Hospital G. Martino, Via Consolare Valeria, 98125 Messina, Italy; 3 Department of Emergency Medicine, University Hospital V. Emanuele, Via S. Sofia, 95123 Catania, Italy; 4 Department of Emergency Medicine, General Hospital S. Elia, Via Luigi Russo, 93100 Caltanissetta, Italy; 5 Sicilian Regional Pharmacovigilance Center, Clinical Pharmacology Unit, University Hospital G. Martino, Via Consolare Valeria, 98125 Messina, Italy

**Keywords:** Emergency department, Adverse drug event, Seriousness, Causality, Preventability

## Abstract

**Background:**

Adverse drug events (ADEs) are a significant cause of emergency department (ED) visits, with a major impact on healthcare resource utilization. A multicentre observational study, aimed to describe frequency, seriousness and preventability of ADEs reported in four EDs, was performed in Sicily (Italy) over a 1-year period.

**Methods:**

Two trained monitors for each ED supported clinicians in identifying ADEs of patients admitted to EDs between June 1st, 2013 and May 31st, 2014 through a systematic interview of patients or their caregivers and with an additional record review. A research team analyzed each case of suspected ADE, to make a causality assessment applying the Naranjo algorithm and a preventability assessment using Schumock and Thornton criteria.

Absolute and percentage frequencies with 95% confidence interval (CI) and medians with interquartile ranges (IQR) were estimated. Logistic regression models were used to evaluate independent predictors of serious and certainly preventable ADEs.

**Results:**

Out of 16,963 ED visits, 575 (3.4%) were associated to ADEs, of which 15.1% resulted in hospitalization. ADEs were classified as probable in 45.9%, possible in 51.7% and definite in 2.4% of the cases. Moreover, ADEs were considered certainly preventable in 12.3%, probably preventable in 58.4%, and not preventable in 29.2% of the cases. Polytherapy influenced the risk to experience a serious, as well as a certainly preventable ADE. Whilst, older age resulted an independent predictor only of serious events. The most common implicated drug classes were antibiotics (34.4%) and anti-inflammatory drugs (22.6%). ADEs due to psycholeptics and antiepileptics resulted preventable in 62.7 and 54.5% of the cases, respectively. Allergic reactions (64%) were the most frequent cause of ADE-related ED visits, followed by neurological effects (10.2%) that resulted preventable in 1.9 and 37.3% of the cases, respectively.

**Conclusion:**

ADEs are a frequent cause of ED visits. The commonly used antibiotics and anti-inflammatory drugs should be carefully managed, as they are widely involved in mild to severe ADEs. Polytherapy is associated with the occurrence of serious, as well as certainly preventable ADEs, while older age only with serious events. A greater sensitivity to drug monitoring programs among health professionals is needed.

## Background

Adverse drug events (ADEs) have a considerable impact on public healthcare and are a significant burden on healthcare resources [1]. Emergency departments (EDs) constitute an essential part of the healthcare system and an important source of information regarding incidence and characteristics of ADEs, as they are an interface between hospitals and communities [2, 3]. Several studies have analysed ED visits potentially related to drug therapy [2, 4–10]. However, a wide range of ADE-related ED visits was reported and available evidence suggests that 0.6–12% of all visits are due to ADEs [5, 7, 10–12]. A recent study based on an active monitoring project regarding ADEs in EDs, carried out in the United States in 2013 and 2014 (*NEISS-CADES, The National Electronic Injury Surveillance System-Cooperative Adverse Drug Event Surveillance*), estimated a rate of 4.0 ED visits per 1000 inhabitants year due to ADEs [9]. The wide range of reported ADE-related ED visits may reflect some methodological variances due to different types of hospital settings, study design, data source, variability in the definition of ADE, difficulty in diagnosis and determination of ADEs [6]. Indeed, many studies conducted in EDs have been limited to one hospital centre [13, 14], a specific population [15, 16], specific therapeutic classes or type of ADEs [17–20]. Other findings are attributable to retrospective study design [21, 22], short periods of observation [2, 5, 6, 23–25], or lack of information on preventability [7, 10, 26]. Moreover, some literature studies reported preventability assessment referred only to therapeutic classes [4, 27, 28]. Even though several studies were made in different European countries, few data are available especially in a South of Italy setting. Thus, more knowledge on occurrence, characteristics and preventability of ADEs is needed. In view of the above findings, the aims of this study were to determine the rate of ADEs leading to ED visits in four hospitals in Sicily (Italy) and to evaluate ADEs’ seriousness and preventability. Furthermore, the drug classes most frequently involved in ADEs, the characteristics of ADEs and their frequency were also evaluated, in terms of both severity and preventability.

## Methods

### Data source and data collection

An active monitoring project of ADEs in four EDs in Sicily (Italy) was carried out in a one-year period. The University Hospitals of Messina and Catania and the General Hospitals *S. Elia* of Caltanissetta and *Villa Sofia-Cervello* of Palermo were selected for this study, as they serve widespread catchment areas of Sicily, which is a large Italian region that includes around 5 million inhabitants.

All patients aged ≥18 presenting to the four EDs between June 1st, 2013 and May 31st, 2014, were eligible for enrolment. For this study data concerning ADE-related ED visits were recorded in a dedicated database. ED records of involved hospitals were also reviewed. Two trained monitors (one pharmacist and one physician) with experience in pharmacovigilance were assigned to each hospital and they supported clinicians in identifying ADEs and gathering all available information through an accurate and systematic interview of patients (or their caregivers). Furthermore, an additional review of patients’ records was performed by monitors to detect other potential missed cases of ADEs that were included only if confirmed by ED physicians. A research group composed of clinical pharmacologists, operating in the Sicilian Pharmacovigilance Centre sited at University Hospital of Messina, ED physicians and monitors revised all detected cases of ADEs. In detail, the team analyzed every case of suspected ADE, to assess the correlation between drug administration and ADE onset using the Naranjo algorithm.

In particular, biological plausibility of symptoms and signs and characteristics of each suspected drug, plausible time relationship between drug intake and symptoms occurrence, potential alternative causes were considered. If a patient was in polytherapy, the association with ADE was evaluated for each drug taken. For each patient the following information were collected: demographic characteristics, clinical status at ED visit, medication use (prescription, over-the-counter, complementary and alternative medications), medical history, previous medication intolerance and allergies, as well as an accurate description of observed symptoms. All data were recorded in a dedicated database.

### Case definition and outcome measurement

The primary outcome of the study was to evaluate the rate of ADEs presenting in EDs. We included all cases of ADE diagnosed by ED physicians, due to prescription drugs, over-the counter medications, dietary supplements, or homeopathic products. An ADE was defined as “an injury resulting from medical intervention related to a drug”, a definition that was intended to encompass harm that arises from medication errors as well as typical adverse drug reactions [29]. Thus, ADEs include all events which derive from appropriate or inappropriate use of a medicinal product within as well as outside the terms of the marketing authorization.

Patients were excluded from the study if (1) they left the ED before being seen by the ED physician; (2) they were seen directly by a consultant specialist physician rather than an ED physician; (3) data collection was not completed; (4) lack of diagnosis by the ED physician or (5) they were paediatric patients (age < 18). The causal relationship between the ADE and the suspected drug was assessed with the Naranjo algorithm, and each ADE case was categorized as: definite (score ≥ 9), probable (scores 5–8), possible (scores 1–4) or doubtful (score ≤ 0) [30]. Preventability of ADEs was also assessed, using the modified Schumock and Thornton criteria [31]. In our study all “certainly preventable” ADEs derived from one of the following suspected causes, according to European Pharmacovigilance guidelines [32]: drug abuse (intentional but excessive use of drug), misuse (intentional and inappropriate use for the patient’s clinical condition, age, weight, dose, route, or frequency of administration, or history of allergies or previous reactions), overdose, medication error.

Serious ADEs were classified as all events resulting as fatal, life-threatening, leading to hospitalization, inducing serious/permanent disability [32]. Drugs involved in ADEs were classified according to the Anatomical Therapeutic Chemical (ATC) classification system [33] and ADEs were coded using the Medical Dictionary for Regulatory Activities (MedDRA®) [34]. Cases were classified by ED physician-developed primary diagnosis and grouped as previously published [9].

### Statistical analysis

In order to assess basal demographic characteristics and drug-related variables of patients with ADEs a descriptive statistical analysis was carried out. We calculated the rate of ADEs leading to ED visits as the ratio between the number of patients admitted to EDs who presented with an ADE and the total number of patients admitted to EDs during the study period. We estimated the rate of hospitalization for ADEs following ED visits by dividing the number of hospitalizations for ADEs by the total number of ADE-related ED visits in the same period. We used absolute and relative frequencies with 95% confidence interval (CI) for categorical variables, and medians with interquartile ranges (IQR) to estimate continuous variables. A non-parametric approach was performed as some of the numerical variables were not normally distributed after applying the Kolmogorov-Smirnov test. The Pearson’s chi-squared test and the Mann-Whitney U test were used to compare subjects’ characteristics, according to ADE seriousness. We applied a univariate logistic regression model to assess the possible influence of predictive factors of serious and certainly preventable ADEs, such as gender, age, number of reported comorbidities, comorbidities index values and number of administered drugs. Patients with not serious ADEs and patients with possibly preventable or not preventable ADEs were used as comparators, respectively. Furthermore, for each analysis the predictors gender, age and number of drugs taken were considered for a multivariate logistic regression model. The number of concomitant diseases and comorbidity index, that are inter-related to number of drugs assumed, were excluded from the analyses in the multivariate model in order to avoid multicollinearity. Crude and adjusted odds ratios (ORs) with 95% confidence intervals (CIs) were calculated for each variable of interest in the univariate and in the multivariate models, respectively. *P* values ≤0.05 were chosen as the threshold of statistical significance. All the analyses were conducted with SPSS.20.0 (IBM Corp. SPSS Statistics).

## Results

### Characteristics of study population

**Fig. 1:**
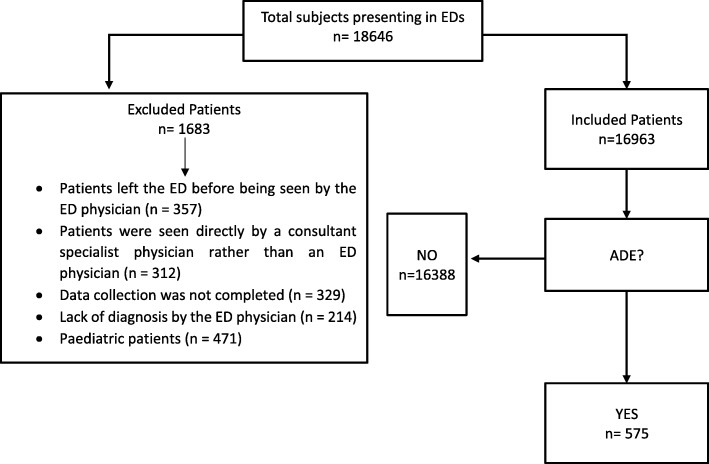
Study flow chart

During the one-year study period, a total of 18,646 patients were admitted to the EDs. Among these, 9.0% were not included (Fig. 1). An ADE was detected in 575 cases, with an overall prevalence rate of 3.4% (95%CI, 3.1–3.7). Among ADE-related visits, 170 (29.6%; 95%CI, 25.8–33.3) were associated with serious events and hospitalization was required in 87 patients (15.1%; 95%CI, 12.2–18.1). Demographic characteristics of patients with ADEs are reported in Table 1. Most patients with ADEs were females (63.1%; 95%CI, 59.2–67.1) and the median age was 52.0 years (IQR: 29.0). Patients with serious ADE were older than patients with not serious ADEs [62.0 years (IQR: 34.0) vs 49.0 years (IQR: 27.0); *p* < 0.001]. In particular, in patients affected by serious ADEs 46.8% were ≥ 65 years, compared to 19.9% without serious ADEs. The median number of drugs taken by patients who developed serious ADEs was higher than patients with not serious ADEs [3.0 (IQR: 4.0) vs 1.0 (IQR: 0.0); p < 0.001].

**Table 1:** Demographic characteristics of patients with adverse drug events (ADEs) leading to Emergency Departments (ED) visits

	ADE-related ED visits	Serious ADE-related ED visits		Not serious ADE-related ED visits		
	No. of Cases (%)	95% CI	No. of Cases (%)	95% CI	No. of Cases (%)	95% CI
Gender						
^a^Males	212 (36.9)	32.9–40.8	59 (34.7)	27.5–41.9	149 (37.2)	32.4–41.9
Females	363 (63.1)	59.2–67.1	111 (65.3)	58.1–72.5	252 (62.8)	58.1–67.6
Patient age group, years						
^b^18–34	118 (20.5)	17.2–23.8	27 (15.6)	10.2–21.0	86 (22.2)	18.1–26.3
^c^35-49	143 (24.1)	20.6–27.5	23 (13.3)	8.2–18.4	118 (28.3)	24.0–32.6
50–64	150 (25.3)	21.8–28.7	39 (22.5)	16.3–28.8	111 (26.6)	22.4–30.9
65–79	116 (19.5)	16.3–22.7	52 (30.1)	23.2–36.9	64 (15.3)	11.9–18.8
≥80	48 (8.1)	5.9–10.3	29 (16.8)	11.2–22.3	19 (4.6)	2.6–6.6
No. of medications by category						
^d^1	380 (66.1)	62.2–70.0	57 (33.5)	26.4–40.6	319 (79.6)	75.6–83.5
≥2	195 (33.9)	30.0–37.8	113 (66.5)	59.4–73.6	85 (20.4)	16.5–24.4
Preventability assessment						
Certainly preventable	71 (12.3)	9.7–15.0	42 (24.7)	18.2–31.2	29 (7.2)	4.7–9.8
Probably preventable^e^	336 (58.4)	54.4–62.5	101 (59.4)	52.0–66.8	232 (57.9)	53.0–62.7
Not-preventable^f^	168 (29.2)	25.5–32.9	27 (15.9)	10.4–21.4	140 (34.9)	30.2–39.6

*95% CI* 95% confidence interval

^a^4 patients with unspecified ADE seriousness

^b^2 patients with unspecified ADE seriousness

^c^2 patients with unspecified ADE seriousness

^d^4 patients with unspecified ADE seriousness

^e^3 patients with unspecified ADE seriousness

^f^1 patient with unspecified ADE seriousness

Using the Naranjo algorithm [31], all included ADEs were classified as probable in 45.9% (95%CI, 41.8–50), possible in 51.7% (95%CI, 47.6–55.7) and definite in 2.4% (95%CI, 1.2–3.7). With regard to preventability, 12.3% (95%CI, 9.7–15.0) of ADEs were considered certainly preventable, while 58.4% (95%CI, 54.4–62.5) were possibly preventable and only 29.2% (95%CI, 25.5–32.9) not preventable. In detail, among 71 cases classified as certainly preventable, 24 cases of ADEs were related to drug abuse, 20 cases to misuse, 17 cases derived from unintentional/intentional overdose and 10 from medication errors (Table 2). Finally, among possibly preventable ADEs, 20 cases related to drug-drug interactions and 1 off-label use occurred.

**Table 2:** Suspected causes for a certainly preventable assessment and drugs involved

Suspected causes (No. of cases)	Drugs involved (No. of cases)^a^
Abuse (24)	Lorazepam (6), quetiapine (3), clonazepam (2), haloperidol (2), bromazepam (2), paroxetine (2), lithium (2), methadone (2), triazolam (1), diazepam (1), chlorpromazine (1), telmisartan (1), alprazolam (1), tosylchloramide (1), clomipramine (1), citalopram (1), promazine (1), acetylsalicylic acid (1), oxazepam (1), venlafaxine (1), delorazepam (1), oxcarbazepine (1), brotizolam (1), valproic acid (1), pregabalin (1), tramadol (1)
Misuse (20)	Digoxin (2), acetylsalicylic acid (2), metformin (2), etoricoxib (2), ceftriaxone (1), promazine (1), clopidogrel (1), doxazosin (1), amoxicillin+clavulanic acid (1), ketoprofen (1), levofloxacin (1),
Overdose (17)	Lorazepam (3), oxcarbazepine (2), carbamazepine (2), warfarin (2), olanzapine (2), acenocoumarol (2), olmesartan (2), morniflumate (1), oxcarbazepine (1), furosemide (1), acetylsalicylic acid (1), nimesulide (1), ketoprofen (1), paracetamol (1), bisoprolol (1), valproic acid (1), tosylchloramide (1), amoxicillin (1), oxycodone+naloxone (1)
Medication error (10)	Warfarin (2), human insulin (1), betamethasone (1), insulin aspart (1), metformin (1), prednisone (1), glicazide (1), paracetamol (1), clonazepam (1), furosemide (1), levothyroxine (1)

^a^The sum of suspected drugs is higher than the total number of cases, since a single patient could have used multiple drugs

As assessed by the multivariate logistic regression models, gender did not influence the risk to experience a serious ADE (OR 1.20; 95% CI 0.78–1.85: *p* = 0.403), as well as a certainly preventable event (OR 1.18; 95% CI 0.70–2.01: *p* = 0.531). Conversely, age ≥ 65 years resulted an independent predictor of serious events (OR 2.66; 95% CI 1.72–4.11: p < 0.001), but not of certainly preventable events (OR 1.20; 95% CI 0.69–2.11: *p* = 0.516). Moreover, polytherapy influenced the risk to experience a serious (OR 6.45; 95% CI 4.26–9.76: p < 0.001), as well as a certainly preventable ADE (OR 1.87; 95% CI 1.11–3.17: *p* = 0.020).

### Drugs associated with adverse drug events

The drug classes most frequently involved in ADEs were antibiotics (34.4%), anti-inflammatory/antirheumatic drugs (22.6%) and antithrombotic agents (9.4%). Serious ADEs were shown in 18.7% (37/198) of ADEs due to antibacterials for systemic use and in 15.4% (20/130) of ADEs related to anti-inflammatory/antirheumatic drugs. ADEs due to diuretics, cardiac drugs (i.e. digital glycosides, antiarrhythmics, cardiac stimulants, vasodilators, other cardiac preparations) and agents acting on the renin-angiotensin system (RAS) were mainly serious (85.7% for diuretics, 84.2% for cardiac drugs, and 85.7% RAS-acting agents). ADEs due to psycholeptics and antiepileptics resulted preventable in 62.7% (32/51) and 54.5% (12/22) of the cases, respectively.

The most commonly involved drugs were amoxicillin/clavulanic acid (14.3%), ketoprofen (10.9%), ceftriaxone (6.6%), amoxicillin (4.9%), and acetylsalicylic acid (4.7%). All 6 cases of ADEs associated with digoxin and 6 out of 7 cases of ADEs related to furosemide and ramipril were classified as serious. ADEs due to lorazepam and warfarin were mainly preventable (9 out of 10 cases for lorazepam and 4 out of 8 cases for warfarin) (Table 3).

**Table 3:** Most commonly implicated drugs in Emergency Department (ED) visits for adverse drug events (ADEs)

		ADE-related ED visits^a,b^	Serious ADE-related ED visits^a,b^	Preventable ADE-related ED visits^a,b^
ATC 5th level	Drug	No. of Cases (%)	No. of Cases (%)	No. of Cases (%)
J01CR02	Amoxicillin/clavulanic acid	82 (14.3)	13 (7.6)	1 (1.4)
M01AE03	Ketoprofen	62 (10.9)	7 (4.1)	2 (2.8)
J01DD04	Ceftriaxone	38 (6.6)	8 (4.7)	1 (1.4)
J01CA04	Amoxicillin	28 (4.9)	4 (2.4)	1 (1.4)
B01AC06	Acetylsalicylic acid	27 (4.7)	9 (5.3)	4 (5.6)
J01MA12	Levofloxacin	17 (3.0)	5 (2.9)	1 (1.4)
M01AB05	Diclofenac	16 (2.9)	1 (0.6)	–
M01AX17	Nimesulide	13 (2.3)	2 (1.2)	1 (1.4)
M01AE01	Ibuprofen	15 (2.6)	2 (1.2)	–
N05BA06	Lorazepam	10 (1.7)	7 (4.1)	9 (12.7)
M01AH05	Etoricoxib	10 (1.7)	3 (1.8)	2 (2.8)
A10BA02	Metformin	9 (1.6)	6 (3.5)	3 (4.2)
H02AB01	Betamethasone	9 (1.6)	1 (0.6)	1 (1.4)
B01AA03	Warfarin	8 (1.4)	5 (2.9)	4 (5.6)
J01MA02	Ciprofloxacin	8 (1.4)	–	–
C03CA01	Furosemide	7 (1.2)	6 (3.5)	2 (2.8)
C09AA05	Ramipril	7 (1.2)	6 (3.5)	–
N02BE01	Paracetamol	6 (1.0)	–	2 (2.8)
N02BE51	Paracetamol, combinations excl. Psycholeptics	6 (1.0)	–	–
N03AF01	Carbamazepine	6 (1.0)	4 (2.4)	2 (2.8)
B01AA07	Acenocoumarol	6 (1.0)	4 (2.4)	2 (2.8)
M03BX05	Thiocolchicoside	6 (1.0)	1 (0.6)	–
C01AA05	Digoxin	6 (1.0)	6 (3.5)	2 (2.8)
N02BA51	Acetylsalicylic acid, combinations excl. psycholeptics	6 (1.0)	–	–

ATC (5th level), Anatomical Therapeutic Chemical classification (5th level)

^a^Drugs implicated in > 5 cases were considered

^b^The sum of suspected drugs is higher than the total number of cases, since a single patient could have used multiple drugs

### Types of adverse drug events

Types of detected ADEs, along with most frequently involved drugs, are summarized in Table 4. The most of ADE related-ED visits were attributed to mild and moderate or severe allergic reactions (64%), certainly preventable in 1.9% (7/368) of the cases and mainly associated with antibiotics and anti-inflammatory/antirheumatic drug administration. Mild to severe neurological effects accounted for 59 ADEs, mainly due to antiepileptics, psycholeptics, and analgesics prescriptions, and 37.3% (22/59) were preventable. Gastrointestinal disturbances occurred in 27 cases, essentially related to anti-inflammatory/antirheumatic drugs, RAS-acting agents and antibiotics and 33.3% (9/27) were preventable.

**Table 4:** Adverse drug events (ADEs) as cause of Emergency Department (ED) visits

ADEs ^a^	No. of Cases (%)	No. of preventable Cases (%)	Most frequent drug classes (No. of Cases)^b^
Mild allergic reaction^c^	214 (37.2)	1 (1.4)	Antibiotics (116), anti-inflammatory/antirheumatic drugs (55)
Moderate to severe allergic reaction^d^	154 (26.8)	6 (8.5)	Antibiotics (64), anti-inflammatory/antirheumatic drugs (52)
Moderate to severe neurological effect^e^	30 (5.2)	12 (16.9)	Psycholeptics (12), antiepileptics (7)
Mild neurological effect^f^	29 (5.0)	10 (14.1)	Psycholeptics (8), analgesics (6)
Gastrointestinal disturbance^g^	27 (4.7)	9 (12.7)	Anti-inflammatory/antirheumatic drugs (10), ^h^RAS-acting agents (3), antibiotics (3), antineoplastic agents (3)
Haemorrhage	21 (3.7)	4 (5.6)	
- Major haemorrhage (i.e. gastrointestinal or pulmonary haemorrhage)	4 (0.7)	1 (1.4)	Antithrombotics (2), RAS-acting agents (1), antianemic preparations (1), beta-blocking agents (1), lipid modifying agents (1), anti-gout preparations (1)
- Minor bleeding (e.g. epistaxis, gingival or conjunctival haemorrhage)	17 (3.0)	3 (4.2)	Antithrombotics (15), lipid modifying agents (1), anti-inflammatory/antirheumatic drugs (1)
Rhythm disorder (e.g. bradycardia, tachycardia, palpitations, atrial fibrillation)	16 (2.9)	3 (4.2)	Cardiac therapy (8), RAS-acting agents (4)
Blood pressure disorder (i.e. hypotension, hypertension)	12 (2.1)	3 (4.2)	Antibiotics (3), cardiac therapy (3), psycholeptics (2), antihypertensives (2), beta blocking agents (2)
Suicide attempt	12 (2.1)	12 (16.9)	Psycholeptics (7), antiepileptics (3), psychoanaleptics (2)
Respiratory distress (e.g. respiratory depression, dyspnoea, desaturation)	7 (1.2)	2 (2.8)	Psycholeptics (4), antibiotics (2)
Hematologic disorder (e.g. anemia, leucopenia, thrombocytopenia)	7 (1.2)	–	Antithrombotics (2), psychoanaleptics (2)
Hyperglycemia	7 (1.2)	3 (4.2)	Anti-diabetes agents (3), corticosteroids for systemic use (2)
Acute renal failure	5 (0.9)	–	Diuretics (3), anti-diabetes agents (2)
Behavioural psychiatric disorder (e.g. anxiety, stupor, aggression)	4 (0.7)	3 (4.2)	Anti-inflammatory/antirheumatic drugs (1), analgesics (1), psycholeptics (1), anti-Parkinson (1)
Rhabdomyolysis	4 (0.7)	–	Antiepileptics (2), RAS-acting agents (1), lipid modifying agents (1), psychoanaleptics (1)
Subcutaneous abscess	4 (0.7)	–	Antibiotics (4)
Pancreatitis	3 (0.5)	1 (1.4)	Antiacid drugs (1), antibiotics (1), anti-inflammatory/antirheumatic drugs (1), calcium channel blockers (1), corticosteroids for systemic use (1), immunosuppressants (1), drugs for bone diseases (1)
Hypoglycaemia	3 (0.5)	2 (2.8)	Anti-diabetes agents (3)
Other effect^i^	16 (2.9)	–	RAS-acting agents (4), psycholeptics (3), diuretics (2), anti-diabetes agents (2), psychoanaleptics (2)

^a^Cases were classified by ED physician-developed primary diagnosis and grouped as published in *NEISS-CADES* analysis. Primary diagnoses are mutually exclusive. For example, an ED visit in which a patient experienced both erythema, dermatitis, pruritus would be categorized as mild allergic reactions; an ED visit in which a patient experienced both vomiting and abdominal pain would be categorized as gastrointestinal disturbance

^b^We have considered only the principal involved drug categories, for each primary diagnosis. In most cases more drug categories were simultaneously involved

^c^Erythema, urticaria, dermatitis, rash, localized or peripheral edema, flushing, pruritus, esanthema

^d^Anaphylaxis, angioedema, facial edema, pharyngeal edema, laryngeal edema, labial edema, eyelid edema, orbital edema, vasculitis, hyperhidrosis, drug hypersensitivity, allergy-related respiratory compromise (dyspnoea, bronchospasm, throat tightness, tachypnea, hyperventilation)

^e^Coma, panic attack, limbs paralysis, cranial traumatism, epilepsy, extrapyramidal disorder, loss of consciousness, headache, syncope, altered mental status

^f^Lethargy, fatigue, drowsiness, asthenia, hypoesthesia, paresthesia, tremor, vertigo

^g^Nausea, vomiting, abdominal pain, epigastric pain, ulcer, erosive gastropathy

^h^RAS-acting agents, agents acting on the renin-angiotensin system

^i^Lactic acidosis (2), limbs phlebitis (2), hypertransaminasemia (2), heart failure (2), dystonia (2), arthralgia (2), jaundice (1), venous sinus thrombosis (1), anemia and peripheral edema (1), conjunctival haemorrhage and epilepsy (1), conjunctival haemorrhage and hypertension (1)

Hospital admission due to ADEs was required in 87 patients and was mainly attributed to allergic reactions (23%). Moderate to severe neurological effects caused 10 hospitalizations. Mild neurological effects, rhythm disorders and haemorrhages accounted for 16.1% of the hospitalizations. Furthermore, 5 cases of acute renal failure, 3 of pancreatitis, and 3 of rhabdomyolysis caused patients’ admissions.

## Discussion

EDs represent a useful setting and a valuable data source to identify the occurrence of ADEs, because of easy access, 24-h availability, and the multidisciplinary nature of consultations [35, 36]. However, a significant heterogeneity among observational studies evaluating ADEs in EDs in terms of observed results and specifically in causality and preventability assessment was shown. The analysis carried out on the basis of real-world data could be essential to further provide additional information on the clinical impact of ED drug-related visits. We think that a focus on severe and preventable ADEs is interesting, in particular because drug classes that will need special monitoring result highlighted from our study. The evaluation of drug classes mainly involved in ADEs is also interesting because of the different results emerging from international and Italian studies. Even though several studies were made in different European countries, few data are available in Italy and especially in a South of Italy setting. In accordance with previous studies [5, 10–12] an overall prevalence rate of 3.3% of ADE-related ED visits was recorded. Our results are in accordance to those stated in a prospective observational study conducted in 22 Italian EDs *(PSADE [ADE in Pronto Soccorso] study),* reporting a 3.3% rate of patients affected by ADE [10]. The ADE-related hospitalization rate in our study was 15.1%. This result is in agreement with two different ED studies in Italy [5, 8]. Conversely, it differs from several international studies [9, 12, 37] in which higher hospitalization rates were observed, such as the *NEISS-CADES* study that reported 27.3% of hospitalizations caused by ADEs [9]. The inclusion of ED observation status or transfers to another facility for acute medical care might partially explain this difference.

It is very difficult to establish a clear cause-effect relationship between drug and adverse event, so clinical evaluations are necessary. We used a standardized causality assessment method, the Naranjo algorithm, to define the probability category for each ADE case. In our study, the frequency of probable ADEs was 45.9%. Various studies reported a wide range of probable ADE frequency: an Australian prospective study found 70.1% of probable ADEs and 24.1% possible, while another study identified 30.8% of probable events and 7.5% possible [15, 38]. This could be justified by differences in the assessment of causality criteria by subjective clinical judgments, usually based on limited clinical data.

In our investigation, ADEs identified in ED visits affected women more frequently, in accordance with the *PSADE* study [10]. Hormonal status, body constitution (body size, body fat), gender differences in drug metabolism and elimination, may influence the probability of experiencing ADEs. Moreover, women are more likely to use several classes of medications and this could explain the different chance of having ADEs between genders [39]. However, gender did not influence the risk to experience a serious, in accordance with a previous study [4]. On the contrary, in the PSADE study, male gender resulted associated with the occurrence of serious ADEs [10].

In our study, the higher rates of serious ADEs were observed in ≥65 years group (46.8%) and in patients treated with more than one drug (66.5%). Polytherapy influenced the risk to experience a serious, as well as a certainly preventable ADE, while age ≥ 65 resulted an independent predictor only of serious events, in accordance with previous studies [4, 8, 10]. It is acknowledged that older age is strongly associated with polytherapy, primarily because of comorbidities (e.g. cardiovascular or renal diseases, hypertension, diabetes) [40], both of which lead a high risk of serious ADEs [41–43]. Moreover, polytherapy could cause a higher risk to develop inappropriateness conditions, classified as certainly preventable events.

The appropriate use of drugs and population characteristics play a key role in the development of ADEs. However, many patients are inappropriately treated [44–53] and available evidence indicates that approximately 50% of ADEs are preventable [54, 55]. In our study, we found a high rate of avoidable ADEs. Overall, about 70% of ADEs were probably (58.4%) or certainly (12.3%) preventable. This finding is in agreement with previous studies [8, 28]. Two prospective, observational studies in EDs identified 70.4 and 68% preventable ADEs [12, 56]. Moreover, data from either retrospective and prospective studies indicate 70% of ED visits as preventable [57]. Similar to other studies [4, 12, 27], our investigation focused on certainly preventable ADEs derived from inappropriate drug use (misuse), abuse, overdose, medication errors. Drug-drug interactions and off-label use were reported among possibly preventable ADEs. A focus on severe and preventable ADEs is interesting because drug classes that will need special monitoring result highlighted from our study. In details, drugs that require constant monitoring due to the risk of acute toxicity (e.g. coumarin anticoagulants, digital glycosides, lithium salts) and central nervous system drugs (i.e. benzodiazepines, psycholeptics, antiepileptics, and psychoanaleptics) have been implicated in abuses, misuses, overdoses. Therefore, additional prevention strategies are needed to improve adherence to medication and the safety of drug prescribing. As previously reported, prevention of ADEs by identifying individuals at high risk is central to improve patient care and outcomes. In particular, additional monitoring and attention towards patients who are at high risk could reduce the impact of ADEs both in terms of cost and quality of care [58, 59].

Antibiotics and anti-inflammatory/antirheumatic drugs, responsible for 57% of overall reports, were the therapeutic subgroups mainly involved in suspected ADEs in our study. This result partially disagrees with the *NEISS-CADES* study, where anticoagulants, antibiotics, diabetes agents, opioid analgesics, and antipsychotics were the most frequent drug classes related to ADE visits [9]. A review of retrospective and prospective observational studies reported that non-steroidal anti-inflammatory drugs, anticonvulsants, antidiabetic agents, antibiotics, respiratory drugs, hormones, central nervous system and cardiovascular drugs were most often implicated in ED visits [57]. However, several Italian studies [7, 10] confirm antibiotics and anti-inflammatory drugs as the therapeutic classes mainly involved in ADEs. These results might be partially explained by the wide use of anti-inflammatory drugs, most of them are available without medical prescription and then without accurate monitoring. Furthermore, in Italy, antibiotics are more widely prescribed among outpatients, often inappropriately, compared to Northern European countries [10, 60]. In accordance with another study, diuretics, cardiac drugs and RAS-acting agents were associated with higher rates of serious ADEs [10]. In fact, these drug groups require careful and constant monitoring for safe use. The most commonly involved drugs were amoxicillin/clavulanic acid, ketoprofen, ceftriaxone, amoxicillin and acetylsalicylic acid. These findings were similar to several studies conducted in outpatient settings [6, 8, 11]. Furthermore, in our study, warfarin was mainly related to certainly preventable ADEs, while digoxin, furosemide, and ramipril to serious events, because of their high toxicity, which requires close monitoring. ADEs frequently recorded in ED visits were mild and moderate to severe allergic reactions (37.2 and 26.8%, respectively), in line with previous data [2, 7, 8, 10]. These results might be partially explained because allergic reactions tend to be frequently recognized and reported by health professionals and, therefore, easily attributable to the previous drug administration. Furthermore, moderate to severe and mild neurological effects involved 5.2 and 5% of ED visits respectively, followed by gastrointestinal disturbances (4.7%). These data are in accordance with the *NEISS-CADES* study, where dermatologic, gastrointestinal and neurological events were the most frequent reported ADEs [11]. Allergic reactions and gastrointestinal disorders were mostly related to antibiotics and anti-inflammatory drugs, while neurological effects were related to psycholeptics, antiepileptics, and analgesics. Moreover, preventability assessment applied to primary diagnosis showed that 37.3% of neurological effects and 33.3% of gastrointestinal disturbances were certainly preventable. Few previous studies evaluated ADEs’ preventability in the context of ED [4, 28], but they only analysed the associated pharmacological categories, and not affected systems or associated diagnosis.

In the FORWARD study, antithrombotics, RAS-agents, NSAIDS, and diuretics were most frequently associated with hospitalizations [27]. Otherwise, in our study, hospitalizations were mainly due to moderate to severe allergic reactions and neurological effects, and the most associated therapeutic classes were antibiotics and psycholeptics and antiepileptics, respectively. Moreover, about 63% of ADEs attributed to psycholeptics and 54.5% related to antiepileptics were certainly preventable. The reduction of preventable ADE-related hospital admissions should be the target of intervention programmes aimed to improve prescriptive appropriateness in general practice. Careful monitoring of commonly used drugs, such as antibiotics, anti-inflammatories and nervous system drugs, could improve patient safety.

This study adds important information to the general knowledge about the impact of ADEs in ED visits. Thanks to ED physician-monitor collaboration, the identification of ADE cases at the time of the access to the ED or through patient record reviews was accurate. This study also has the advantage of ADE preventability assessment; indeed, about 12.3% of the cases resulted “certainly” preventable. However, preventability assessment using the modified Schumock and Thornton criteria has several potential limitations that need to be considered, especially related to missing anamnestic information in patient records. This could underestimate ADE identification and influence the causality assessment procedure. A recent study compared different methods for determining preventability ADEs in EDs claiming that a “best practice-based” preventability assessment was to be preferred by clinicians over an “algorithm-based” approach like the modified Schumock and Thornton criteria. The “best practice-based” approach required in any case a high level of clinical experience and expertise to assess an overall preventability ADEs [61]. Nevertheless, pharmacists or physicians were involved in our study, as monitors in EDs, to help ED physicians in obtaining medication histories, monitoring polypharmacy, and collecting additional information for causality and preventability assessment and to develop this critical component of patients’ interview.

Furthermore, the number of analysed predictors of severity and preventability of ADEs is limited and more variables could influence the occurrence of events. Drug consumption in the general population also influences ADE occurrence; for example, the wide use of anti-inflammatory drugs in self-medication and the frequent overuse of antibiotics in Italy, could have influenced the higher rate of ADEs related to these drug categories.

## Conclusion

The results from this study highlight the need to promote appropriate education strategies, aimed to improve awareness of pharmacovigilance. The first approach involves focusing on the analysis of the process of care, while the second method is through identification of patients who are ‘at-risk’, as elderly subjects, patients in polytherapy, and with comorbidities. Polytherapy is associated with the occurrence of serious, as well as certainly preventable ADEs, whilst older age only with serious events, providing a strong rationale to improve safety and to obtain greater sensitivity to drug monitoring programs among health professionals. The analysis carried out on the basis of real-world data could be essential to further develop interventions designed to measurably reduce preventable harm from medications. Most preventable ADEs involved two classes of drugs, psycholeptics and antiepileptics, widely used and sometimes inappropriately used. The heavy burden of preventable ADEs may translate into potentially significant cost savings if these education strategies can be implemented further.

## Abbreviations

ADE: Adverse drug event
ADR: Adverse drug reaction
ATC: Anatomical therapeutic chemical
CI: Confidence interval
ED: Emergency department
IQR: Interquartile range
MedDRA: Medical dictionary for regulatory activities
RAS: Renin-angiotensin system
SPC: Summary of product characteristics
